# An Association Between Possible Sarcopenia as an Early Marker and Mild Cognitive Impairment: A Cross-Sectional Study

**DOI:** 10.3390/healthcare13161963

**Published:** 2025-08-11

**Authors:** Junga Lee, Guang-Lei Zhang

**Affiliations:** Graduate School of Sports Medicine and Science, Kyung Hee University, Yongin-si 17104, Gyeonggi-do, Republic of Korea; guangleizhang@khu.ac.kr

**Keywords:** sarcopenia, mild cognitive impairment, older adults

## Abstract

**Background/Objectives**: The purpose of this study is to investigate the association between possible sarcopenia, considered as an early marker before its diagnosis, and mild cognitive impairment. **Methods**: A cross-sectional study was conducted involving 60 physically inactive participants aged 40–69. Assessments included body composition, physical activity, and cognitive evaluations. The association between sarcopenia and mild cognitive impairment was examined using binary logistic regression, with odds ratios (ORs) and 95% confidence intervals (CIs) reported. **Results**: Participants with possible sarcopenia were found to have a 12.25 times higher risk of developing mild cognitive impairment (OR: 12.250; 95% CI: 1.692–88.711; *p* = 0.013). **Conclusions**: The findings highlight the critical role of early diagnosis of and intervention in sarcopenia to prevent cognitive decline. Future longitudinal studies are necessary to clarify causal relationships and optimize preventive strategies.

## 1. Introduction

Individuals aged 65 and over accounted for approximately 9.3% of the world population in 2020, and this proportion is expected to rise to about 16% by 2050 [1]. South Korea and Japan are experiencing rapid aging, with projections indicating that by 2025, the proportion of people aged 65 and over will reach approximately 20.3% in South Korea and about 30% in Japan [2]. Older adults are increasingly facing age-related diseases, particularly sarcopenia and mild cognitive impairment, which have emerged as significant health concerns. Sarcopenia affects approximately 10% to 27% of individuals aged 65 and older [3]. Mild cognitive impairment occurs in about 15–20% of the older adults and poses a risk of progression to dementia [4].

Sarcopenia is defined as a decrease in muscle mass and strength, representing a major health issue associated with aging. It is linked to falls, fractures, functional decline, and increased mortality [3,5]. Several physiological mechanisms of sarcopenia include reduced muscle protein synthesis, hormonal changes (e.g., decreased growth hormone and testosterone), increased inflammation, and weakened neuromuscular connections [6,7,8,9]. The Asian Working Group for Sarcopenia (AWGS) recommends criteria for diagnosing sarcopenia, including grip strength (men < 26 kg, women < 18 kg), walking speed (<1.0 m/s), and muscle mass (BMI-adjusted muscle mass: men < 7.0 kg/m^2^, women < 5.7 kg/m^2^) [10]. The European Working Group on Sarcopenia in Older People (EWGSOP) highlights the importance of assessing not only muscle mass but also muscle function. They use specific criteria, including grip strength (less than 27 kg for men and less than 16 kg for women) and walking speed (0.8 m per second or slower). Muscle mass is evaluated through Dual-Energy X-ray Absorptiometry (DXA) or bioelectrical impedance analysis (BIA) [5]. Other research describes sarcopenia as an often-overlooked condition in older adults, highlighting the importance of muscle strength and walking ability and identifying physical inactivity and nutritional imbalance as contributing factors [11]. These studies advocate for a comprehensive diagnostic approach to sarcopenia and emphasize the need for early diagnosis and intervention. Establishing criteria for sarcopenia as an early marker, prior to its diagnosis, would be helpful in preventing the condition.

Mild cognitive impairment, another condition affecting the daily lives of older adults, is characterized by slight cognitive decline. Mild cognitive impairment increases the risk of progression to dementia, potentially impairing independent living [4], and is associated with structural changes in the brain, such as hippocampal atrophy and amyloid plaque accumulation, leading to memory and other cognitive deficits [12,13,14]. The assessment of mild cognitive impairment primarily uses the Mini-Mental State Examination (MMSE) and the Montreal Cognitive Assessment (MoCA) [15]. Furthermore, the diagnosis of mild cognitive impairment involves complex and comprehensive methods such as brain imaging and cerebrospinal fluid analysis [16,17,18]. Mild cognitive impairment also requires the establishment of criteria for preventive measures prior to diagnosis, which can aid in prevention.

These conditions, including sarcopenia and mild cognitive impairment, not only significantly reduce the quality of life for the older adults but also impose substantial burdens on healthcare systems. Managing and preventing sarcopenia and mild cognitive impairment are critical tasks in older adults’ healthcare within aging societies. Research on the relationship between sarcopenia and mild cognitive impairment is ongoing, with recent studies indicating that older adults with sarcopenia are at a higher risk of experiencing mild cognitive impairment [19,20]. A previous meta-analysis including seven cross-sectional studies found a significant association between sarcopenia and cognitive impairment [19]. Another previous meta-analysis that included 13 studies and 27,428 participants found that the pooled prevalence of mild cognitive impairment among individuals with sarcopenia was 20.5%, indicating a relatively high prevalence of mild cognitive impairment in people with sarcopenia [21]. This may be due to interactions between muscle and brain, where muscle activity could have neuroprotective effects.

Previous studies suggest that the earlier sarcopenia occurs, the higher the risk of developing mild cognitive impairment, and this association was also observed in middle-aged populations [22,23]. A previous study that included 1175 older adults without dementia and followed them for an average of 5.6 years found that a higher degree of sarcopenia at baseline was associated with an increased risk of developing Alzheimer’s dementia and mild cognitive impairment, as well as a faster rate of cognitive decline [22]. Another longitudinal study involving 496 adults aged 50 years and older found a significant association between sarcopenia and mild cognitive impairment, and the annual increase in the prevalence of mild cognitive impairment was higher among individuals with sarcopenia (1.5%) compared to those without sarcopenia (0.8%) [23]. Not only sarcopenia but also possible sarcopenia has been significantly associated with mild cognitive impairment [24,25]. A previous cross-sectional study involving 1394 older adults found that possible sarcopenia was significantly associated with an increased risk of mild cognitive impairment [24]. Furthermore, a recent meta-analysis of 31 studies demonstrated that both sarcopenia and possible sarcopenia were significantly linked to mild cognitive impairment [25]. The early identification of possible sarcopenia and mild cognitive impairment may be crucial in preventing the development of these conditions. Understanding the associations between sarcopenia and mild cognitive impairment and establishing preventive criteria could be effective in reducing the risks associated with both conditions. Therefore, the purpose of this study is to investigate the association between sarcopenia, considered a more effective early marker, and mild cognitive impairment.

## 2. Materials and Methods

The association between possible sarcopenia, as an early marker, and mild cognitive impairment was examined in a cross-sectional study. The required sample size was estimated using G*Power 3.1 for a binary logistic regression analysis. The calculation assumed a two-tailed test, an alpha level of 0.05, a power of 0.80, and an expected odds ratio (OR) of 5.1, based on a previously published study [26]. This study comprised 60 physically inactive participants, equally divided by gender across three age brackets: 40–49, 50–59, and 60–69 years. To ensure gender balance among middle-aged and older participants, each age group included 10 men and 10 women. Participants with recognized physical disabilities or chronic health issues impacting mobility were not included. Recruitment of participants took place in Y City, South Korea, via poster advertisements displayed in community venues. The study protocol was approved by the Institutional Review Board (IRB) of K University. Participants were informed of their right to withdraw from the study at any time without incurring any disadvantages. Strict confidentiality was maintained to protect the privacy of the participants. All measurement results relevant to the study were promptly communicated to the individuals involved.

### 2.1. Measurements

Body height was accurately measured to the nearest 0.1 cm using an ultrasonic device (GL-150Tech, G-Tech International, Uijeongbu, Republic of Korea). Body weight, fat mass, fat percentage, and skeletal muscle mass were assessed with a bioelectrical impedance analysis device (Inbody 270, Inbody, Seoul, Republic of Korea).

Participants had their waist circumference measured while standing with feet shoulder-width apart using a non-elastic tape to find the midpoint between the lower rib and upper iliac crest at the end of expiration. The hip circumference of participants was measured with their feet together, recording the maximum circumference around the buttocks. The waist and hip circumferences of participants were each measured twice, and the average values were calculated.

An assessment of blood pressure and resting heart rate was conducted using a blood pressure monitor (Model JPN710T, OMRON, Shanghai, China). Accuracy was ensured by taking these measurements three times and calculating the average value.

Physical activity levels and sedentary time were assessed using the Korean Version of the International Physical Activity Questionnaire (IPAQ) Short Form. This questionnaire asked about the frequency and duration of vigorous-intensity activities, moderate-intensity activities, walking, and sedentary time during the past seven days. According to the IPAQ scoring protocol, participants with a total physical activity level of less than 600 MET-minutes per week are classified as physically inactive [10].

Measurements of maximal oxygen uptake, resting metabolic rate, and predicted resting metabolic rate were conducted with a wireless gas analyzer (K5, Cosmed, Albano Laziale, Italy).

Mild cognitive impairment was evaluated with the Korean version of the Mini-Mental State Examination, which has a total score of 30 points [27]. A score below 27 was used to suggest mild cognitive impairment [28].

The criteria for identifying possible sarcopenia align with the 2019 guidelines established by the Asian Working Group for Sarcopenia, which indicate possible sarcopenia with a calf circumference of less than 34 cm for men and less than 33 cm for women; grip strength under 28 kg for men and under 18 kg for women; or a five-time chair stand test duration of 12 s or more [10].

Indicators for potential sarcopenia were adjusted to identify early markers as follows: a calf circumference of less than 36 cm for men and less than 35 cm for women; grip strength under 32 kg for men and under 22 kg for women; and a five-time chair stand test duration of 10 s or more.

### 2.2. Statistical Analysis

Continuous variables were reported as mean ± standard deviation (SD), and categorical variables were presented as frequencies and percentages. Data normality was tested using the Kolmogorov–Smirnov test. The association between sarcopenia and mild cognitive impairment was analyzed using binary logistic regression, with odds ratios (ORs) and 95% confidence intervals (CIs) provided. Statistical significance was set at *p* < 0.05, and all analyses were conducted using SPSS 23.0 (Chicago, IL, USA).

## 3. Results

There were 60 participants in the study, equally divided into 30 males and 30 females, with an average age of 54.23 years. Detailed characteristics of the participants are provided in Table 1. The present study also reported the basic characteristics of individuals with non-sarcopenia and/or mild cognitive impairment and sarcopenia and/or mild cognitive impairment in Supplementary Table S1.

### Association Between Possible Sarcopenia and Mild Cognitive Impairment

Participants with possible sarcopenia showed a 12.25-fold increased risk of developing mild cognitive impairment compared to those without possible sarcopenia (OR: 12.250; 95% CI: 1.692–88.711; *p* = 0.013). This association remained significant even after adjusting for age (OR: 10.266; 95% CI: 1.355–77.172; *p* = 0.024), as presented in Table 2. The absolute values of all independent variables’ variance inflation factors (VIFs) were less than 2.

## 4. Discussion

This study investigated the association between possible sarcopenia as an early marker and mild cognitive impairment. It revealed that individuals with possible sarcopenia faced a 12.25-fold increased risk of developing mild cognitive impairment. This finding suggests that older adults with sarcopenia are at a notably higher risk of experiencing mild cognitive impairment, confirming a strong association between sarcopenia and cognitive impairment.

These findings emphasize that sarcopenia is closely related not only to physical function decline but also to cognitive function decline. Consistent with previous studies, our study also confirms that sarcopenia significantly impacts both physical and cognitive health [19,29]. Further research suggests that sarcopenia may increase the risk of cognitive decline and dementia, highlighting the importance of early diagnosis and management of sarcopenia [23,30,31].

This association can be explained by several physiological mechanisms. First, sarcopenia is linked to a chronic inflammatory state, which is also associated with cognitive decline [19]. Inflammatory cytokines (e.g., IL-6, TNF-α) can negatively affect neuronal function, leading to cognitive decline [32]. A previous study reported that older adults with increased inflammatory markers have a higher risk of cognitive decline [33]. Second, sarcopenia is related to decreased levels of hormones such as testosterone and growth hormone, which have neuroprotective effects; thus, their reduction can lead to cognitive decline. Another study found that men with lower testosterone levels have a higher risk of cognitive decline [34]. Lastly, sarcopenia can lead to neuromuscular dysfunction, resulting in decreased neural transmission speed and delayed muscle response time. These neuromuscular changes may also be related to cognitive decline. Research has shown that reduced muscle mass and neuromuscular function are associated with cognitive decline [35,36]. Additionally, sarcopenia leads to reduced physical activity, which can decrease brain blood flow and oxygen supply, potentially causing cognitive decline [31,37,38]. Activity plays a crucial role in maintaining brain health.

Regular physical activity and exercise have a direct impact on increasing muscle mass, enhancing strength, and improving cognitive function. A previous study reported that resistance and aerobic exercises are effective in preventing sarcopenia and maintaining cognitive function [39]. In particular, resistance exercise promotes muscle protein synthesis, playing a vital role in maintaining muscle mass [40]. Aerobic exercise improves cardiorespiratory endurance, optimizing oxygen supply throughout the body, which positively affects muscle health and brain function. Another study demonstrated that aerobic exercise leads to structural changes and functional improvements in the brain, enhancing cognitive function [41]. Additional research indicated that aerobic exercise increases hippocampal volume, contributing to improved memory [42]. Previous studies suggest that older adults with higher maximal oxygen uptake (VO_2_max) have a lower risk of sarcopenia and cognitive impairment, emphasizing the importance of cardiorespiratory endurance [43]. This supports the need for exercise programs that increase VO_2_max to maintain overall physical health and cognitive function. Recent research also confirms the association between VO_2_max and sarcopenia and cognitive impairment, highlighting that improved cardiorespiratory endurance can contribute to preventing age-related diseases [31,44,45].

Resistance exercise is effective in increasing strength and muscle mass, playing a crucial role in preventing sarcopenia. A previous study reported that resistance exercise positively impacts muscle function and body composition in older adults [46]. Another study suggested that resistance exercise also positively influences cognitive function [47,48,49]. Such exercises promote neurogenesis and enhance synaptic plasticity, contributing to cognitive function enhancement. Additionally, a different study found that combined exercise programs, which include both resistance and aerobic exercises, are more effective in preventing sarcopenia and cognitive impairment [50]. This integrated approach with various exercise forms is effective in preventing age-related diseases.

Previous studies have shown that lower maximal oxygen consumption is associated with an increased risk of sarcopenia, as reduced cardiorespiratory fitness is often accompanied by decreased physical activity and loss of muscle mass [51,52]. Lower aerobic capacity is also linked to cognitive decline, potentially through mechanisms such as reduced cerebral oxygen supply and impaired neuroplasticity, which may exacerbate the development of cognitive impairment [53,54]. Resting metabolic rate reflects the basal level of energy expenditure, and its decline may be closely related to decreased muscle mass and cardiorespiratory fitness [55,56]. These parameters not only serve as indicators of physical fitness but also play a potential role in the pathophysiology of both sarcopenia and mild cognitive impairment.

This study highlights the clinical significance of possible sarcopenia. Physical and cognitive functions are often already impaired when sarcopenia is clinically diagnosed in older adults [57,58]. Early identification of individuals with possible sarcopenia and the implementation of appropriate interventions may contribute to delaying disease progression and enhancing quality of life [57]. In contrast to prior studies that primarily examined older adults with confirmed sarcopenia, the present study investigates the association between possible sarcopenia and cognitive impairment in physically inactive individuals, providing additional evidence to propose early marker criteria for the prevention of mild cognitive impairment associated with sarcopenia in adults aged 40 to 69 years.

This study has several limitations. First, as a cross-sectional study, it is difficult to establish causality. While useful for identifying associations between sarcopenia and cognitive impairment, it is limited in establishing causal relationships. Future research should focus on long-term longitudinal studies and explore comprehensive health management strategies, including physical activity and nutritional status. Second, this study included only physically inactive individuals to increase the likelihood of detecting potential sarcopenia and mild cognitive impairment in adults aged 40 to 60. This may limit the applicability of the findings to the general population. Recruitment through community poster advertisements may have introduced selection bias, as more health-conscious individuals are more inclined to participate. Additionally, physical inactivity was assessed via self-report, which may involve some reporting bias. Future research should include participants with varying activity levels and adopt more objective assessment methods. Third, education level was not included as a covariate or explanatory factor when assessing cognitive function using the Mini-Mental State Examination. This may have affected the accuracy of the cognitive assessment results. Additionally, basic research is needed to better understand the complex interactions between sarcopenia and cognitive function through various physiological mechanisms.

## 5. Conclusions

In conclusion, this study provides essential foundational data for understanding the relationship between sarcopenia and mild cognitive impairment. It emphasizes the importance of managing muscle function and mass as key risk factors and preventive strategies for both sarcopenia prevention and cognitive function enhancement. These findings contribute significantly to the health management of older adults.

## Figures and Tables

**Table 1:** Basic characteristics of participants.

	Total (*n* = 60)	Total Male (*n* = 30)	Total Female (*n* = 30)	Male 40–49 (*n* = 10)	Female 40–49 (*n* = 10)	Male 50–59 (*n* = 10)	Female 50–59 (*n* = 10)	Male 60–69 (*n* = 10)	Female 60–69 (*n* = 10)
Age (year)	54.23 ± 7.33	54.40 ± 6.99	54.07 ± 7.79	47.10 ± 1.59	45.20 ± 3.26	53.10 ± 2.38	54.20 ± 2.90	63.00 ± 2.40	62.80 ± 2.04
Body weight (kg)	66.44 ± 14.27	74.56 ± 13.02	58.31 ± 10.40	76.12 ± 14.77	61.30 ± 13.85	76.15 ± 16.28	58.55 ± 10.03	71.42 ± 6.84	55.07 ± 5.89
Height (cm)	1.66 ± 0.08	1.73 ± 0.06	1.60 ± 0.05	1.74 ± 0.06	1.61 ± 0.03	1.73 ± 0.06	1.60 ± 0.05	1.71 ± 0.06	1.59 ± 0.04
Body mass index (kg/m^2^)	23.86 ± 3.99	24.98 ± 3.79	22.75 ± 3.94	25.18 ± 3.99	23.75 ± 5.43	25.29 ± 5.06	22.77 ± 3.31	24.45 ± 2.07	21.72 ± 2.69
Waist circumference (cm)	88.75 ± 10.84	92.09 ± 9.91	85.41 ± 10.85	92.39 ± 12.21	87.05 ± 13.98	93.22 ± 12.34	85.75 ± 11.07	90.66 ± 3.38	83.43 ± 7.33
Hip circumference (cm)	98.33 ± 7.21	100.18 ± 6.08	96.47 ± 7.85	100.63 ± 6.60	98.53 ± 9.97	101.70 ± 7.59	97.10 ± 8.04	98.20 ± 3.26	93.79 ± 4.59
Waist-to-hip ratio	0.90 ± 0.06	0.92 ± 0.05	0.88 ± 0.05	0.91 ± 0.06	0.88 ± 0.07	0.91 ± 0.07	0.88 ± 0.05	0.92 ± 0.03	0.89 ± 0.04
Muscle mass (kg)	26.79 ± 6.22	32.00 ± 4.08	21.59 ± 2.48	32.80 ± 4.07	22.43 ± 3.01	32.14 ± 5.39	21.66 ± 2.23	31.05 ± 2.49	20.68 ± 2.04
Fat mass (kg)	18.10 ± 7.48	17.73 ± 7.39	18.47 ± 7.67	17.86 ± 8.53	20.06 ± 10.67	19.15 ± 9.24	18.61 ± 7.12	16.19 ± 3.59	16.73 ± 4.34
Body fat percentage (%)	26.82 ± 7.63	22.93 ± 6.30	30.71 ± 6.88	22.35 ± 7.33	31.17 ± 8.80	23.96 ± 7.95	30.87 ± 6.76	22.48 ± 3.02	30.10 ± 5.36
Resting heart rate (bpm)	70.70 ± 9.23	70.80 ± 8.88	70.60 ± 9.71	67.60 ± 7.28	72.40 ± 12.17	74.10 ± 7.33	72.10 ± 9.12	70.70 ± 11.15	67.30 ± 7.39
Systolic blood pressure (mmHg)	126.55 ± 16.45	130.40 ± 13.93	122.70 ± 18.06	134.00 ± 7.23	116.50 ± 18.99	123.80 ± 14.09	125.70 ± 18.57	133.40 ± 17.35	125.90 ± 16.77
Diastolic blood pressure (mmHg)	81.12 ± 10.69	85.37 ± 8.70	76.87 ± 10.92	87.60 ± 4.74	75.40 ± 14.10	82.40 ± 11.46	77.10 ± 11.51	85.70 ± 8.00	78.10 ± 6.98
Maximal oxygen consumption (ml/min/kg)	23.80 ± 5.50	27.80 ± 4.20	19.79 ± 3.27	29.45 ± 4.81	20.06 ± 4.01	27.72 ± 4.79	19.86 ± 3.67	26.23 ± 2.20	19.46 ± 2.16
Resting metabolic rate (kcal/day)	1980.38 ± 394.38	2188.73 ± 353.36	1772.03 ± 319.00	2274.00 ± 367.60	1931.00 ± 361.87	2121.60 ± 373.16	1640.60 ± 297.54	2170.60 ± 338.39	1744.50 ± 245.55
Predicted resting metabolic rate (kcal/day)	1421.90 ± 238.43	1587.70 ± 212.29	1256.10 ± 117.09	1662.80 ± 228.96	1327.70 ± 136.62	1622.10 ± 245.01	1257.70 ± 100.37	1478.20 ± 108.91	1182.90 ± 61.54
Physical activity (minutes/week)	593.75 ± 752.01	592.67 ± 643.17	594.83 ± 858.743	322.00 ± 240.04	529.00 ± 551.98	600.00 ± 879.56	708.00 ± 1251.96	856.00 ± 585.80	547.50 ± 693.43
Sedentary time (minutes/week)	315.50 ± 181.64	340.00 ± 169.77	291.00 ± 192.52	504.00 ± 165.41	336.00 ± 165.41	291.00 ± 103.97	336.00 ± 273.06	225.00 ± 86.31	201.00 ± 63.33

The Kolmogorov–Smirnov test was applied to assess whether the data conformed to a normal distribution.

**Table 2:** The association between possible sarcopenia and mild cognitive impairment.

	Modal 1	Modal 2
Reference	1	1
Odds ratio (95% CI)	12.250 (1.692, 88.711)	10.266 (1.355, 77.172)
*p*	0.013	0.024
VIF	1.000	1.090

Modal 1 represents the unadjusted logistic regression analysis. Modal 2 represents the logistic regression analysis adjusted for age. VIF: variance inflation factor.

## Data Availability

The original contributions presented in this study are included in the article. Further inquiries can be directed to the corresponding author.

## Supplementary Materials

The following supporting information can be downloaded at https://www.mdpi.com/article/10.3390/healthcare13161963/s1: Table S1: Basic Characteristics of Participants.
