# Supplementary material for: An Association Between Possible Sarcopenia as an Early Marker and Mild Cognitive Impairment: A Cross-Sectional Study
Source: Healthcare (Basel). 2025 Aug 11;13(16):1963. doi: 10.3390/healthcare13161963 (PMC12385444; doi:10.3390/healthcare13161963)
Supplement: Supplementary file 1 [file healthcare-13-01963-s001.zip › healthcare-3699824-supplementary.pdf]

Supplementary Material

Table S1. Basic Characteristics of Participants.

	**Total (n = 60)**	**Possible sarcopenia and/or mild cognitive impairment (n = 8)**	**Non-possible sarcopenia and/or mild cognitive impairment (n = 52)**
Age (year)	54.23 ± 7.33	57.63 ± 7.89	53.71 ± 7.18
Body weight (kg)	66.44 ± 14.27	58.04 ± 11.53	67.73 ± 14.31
Height (cm)	1.66 ± 0.08	1.65 ± 0.08	1.66 ± 0.08
Body mass index (kg/m^2)	23.86 ± 3.99	21.08 ± 2.61	24.29 ± 4.01
Waist circumference (cm)	88.75 ± 10.84	80.50 ± 7.84	90.02 ± 10.73
Hip circumference (cm)	98.33 ± 7.21	92.85 ± 4.35	99.17 ± 7.21
Waist-to-hip ratio	0.90 ± 0.06	0.87 ± 0.05	0.91 ± 0.05
Muscle mass (kg)	26.79 ± 6.22	24.45 ± 5.95	27.15 ± 6.24
Fat mass (kg)	18.10 ± 7.48	13.62 ± 3.98	18.79 ± 7.67
Body fat percentage (%)	26.82 ± 7.63	23.61 ± 6.36	27.32 ± 7.74
Resting heart rate (bpm)	70.70 ± 9.23	70.13 ± 10.40	70.79 ± 9.15
Systolic blood pressure (mmHg)	126.55 ± 16.45	121.25 ± 5.92	127.37 ± 17.41
Diastolic blood pressure (mmHg)	81.12 ± 10.69	78.50 ± 6.93	81.44 ± 11.04
Maximal Oxygen Consumption (ml/min/kg)	23.80 ± 5.50	24.99 ± 4.82	23.61 ± 5.61
Resting metabolic rate (kcal/day)	1980.38 ± 394.38	1897.50 ± 388.26	1993.13 ± 397.49
Predicted resting metabolic rate (kcal/day)	1421.90 ± 238.43	1299.37 ± 174.87	1440.75 ± 242.59
Physical activity (minutes/week)	593.75 ± 752.01	510.63 ± 611.61	606.54 ± 775.66
Sedentary time (minutes/week)	315.50 ± 181.64	228.75 ± 153.76	328.85 ± 183.20
